# Endoscopic urethrotomy versus open urethroplasty for men with bulbar urethral stricture: the OPEN randomised trial cost-effectiveness analysis

**DOI:** 10.1186/s12894-021-00836-1

**Published:** 2021-05-03

**Authors:** Jing Shen, Luke Vale, Beatriz Goulao, Paul Whybrow, Stephen Payne, Nick Watkin, OPEN trial investigators, Trevor Dorkin, Nick Watkin, Anthony Mundy, Paul Anderson, Suzie Venn, Ian Eardley, David Dickerson, Nikesh Thiruchelvam, Richard Inman, Chris Chapple, Andrew Baird, Andrew Sinclair, Rajeshwar Krishnanm, Rowland Rees, James N’dow, Bruce Montgomery, Michael Swinn, Alastair Henderson, John Donohue, Suzie Venn, Robert Mason, Sanjeev Madaan, Mustafa Hilmy, Vivienne Kirchin, Kim Davenport, John McGrath, Tim Porter, Ruaraidh MacDonagh, Amerdip Birring, Ramachandran Ravi, Jawad Husain, Maj Shabbir, Omer Baldo, Sadhanshu Chitale, Mary Garthwaite, Shalom Srirangam, Liaqat Chowoo, Tina Rashid, Rob Skyrme, Jon Featherstone, Ammar Alhasso, Oleg Tatarov

**Affiliations:** 1 Population Health Sciences Institute, Newcastle University, Newcastle upon Tyne, UK; 2 Health Services Research Unit, University of Aberdeen, Aberdeen, UK; 3 Hull York Medical School, University of Hull, Hull, UK; 4 Central Manchester Hospitals NHS Foundation Trust, Manchester, UK; 5 Department of Urology, St George’s University of London, London, UK

**Keywords:** Cost-effectiveness, Economic model, Randomised controlled trial, Urethral stricture, Urethroplasty, Urethrotomy

## Abstract

**Background:**

Bulbar urethral stricture is a common cause for urinary symptoms in men and its two main treatment options both have drawbacks with little evidence on their relative cost-effectiveness. Current guidelines on the management of recurrent bulbar urethral stricture have been predominantly based on expert opinion and panel consensus.

**Objective:**

To assess the relative cost-effectiveness of open urethroplasty and endoscopic urethrotomy as treatment for recurrent urethral stricture in men.

**Methods:**

Set in the UK National Health Service with recruitment from 38 hospital sites, a randomised controlled trial of open urethroplasty and endoscopic urethrotomy with 6-monthly follow-up over 24 months was conducted. Two hundred and twenty-two men requiring operative treatment for recurrence of bulbar urethral stricture and having had at least one previous intervention for stricture were recruited. Effectiveness was measured by quality- adjusted life years (QALYs) derived from EQ-5D 5L. Cost-effectiveness was measured by the incremental cost per QALY gained over 24 months using a within trial analysis and a Markov model with a 10-year time horizon.

**Results:**

In the within trial, urethroplasty cost on average more than urethrotomy (cost difference: £2148 [95% CI 689, 3606]) and resulted in a similar number of QALYs on average (QALY difference: − 0.01 [95% CI − 0.17, 0.14)] over 24 months. The Markov model produced similar results. Sensitivity analyses using multiple imputation, suggested that the results were robust, despite observed missing data.

**Conclusions:**

Based on current practice and evidence, urethrotomy is a cost-effective treatment compared with urethroplasty.

**Keypoints:**

Urethrotomy and urethroplasty both led to symptom improvement for men with bulbar urethral stricture—a common cause for urinary symptoms in men; Urethroplasty appeared unlikely to offer good value for money compared to urethrotomy based on current evidence.

*Trial registration*: ISRCTN: 98009168 (date: 29 November 2012) and it is also in the UK NIHR Portfolio (reference 13507).

*Trial protocol*: The latest version (1.8) of the full protocol is available at: www.journalslibrary.nihr.ac.uk/programmes/hta/105723/#/ and a published version is also available: Stephenson R, Carnell S, Johnson N, Brown R, Wilkinson J, Mundy A, et al. Open urethroplasty versus endoscopic urethrotomy—clarifying the management of men with recurrent urethral stricture (the OPEN trial): study protocol for a randomised controlled trial. Trials 2015;16:600. https://doi.org/10.1186/s13063-015-1120-4.

*Trial main clinical results publication*: Goulao B, Carnell S, Shen J, MacLennan G, Norrie J, Cook J, et al. Surgical Treatment for Recurrent Bulbar Urethral Stricture: A Randomised Open-label Superiority Trial of Open Urethroplasty Versus Endoscopic Urethrotomy (the OPEN Trial), European Urology, Volume 78, Issue 4, 2020, Pages 572–580.

**Supplementary Information:**

The online version contains supplementary material available at 10.1186/s12894-021-00836-1.

## Introduction

Bulbar urethral stricture is a common cause for urinary symptoms (typically difficulty in passing urine) in men. Initial treatment is usually by endoscopic urethrotomy, a procedure that produces widening of the narrowed urethral segment by incising the stricture internally under vision. In about 50% of the cases the stricture will recur requiring re-treatment [1]. This can be via a repeat endoscopic urethrotomy, graduated dilatation or formally repaired by urethroplasty with either excision of the stricture and anastomosis, or augmentation using a graft such as buccal mucosa.

Urethrotomy is most commonly performed for recurrent bulbar stricture because it is minimally invasive, does not require specialist surgical expertise, and has a short period of urethral catheterisation and recovery. However, further recurrence is likely [2]. Open urethroplasty is more invasive, requires specialist expertise, a longer period of catheterisation and a more protracted return to normal activities. Nevertheless, urethroplasty may offer the prospect of long-term cure without the need for further interventions [3, 4]. When men are choosing between these two options, they have to make a trade-off between the invasiveness and effectiveness of each operation [1, 5–8]. Current decision-making is therefore determined by the availability of local expertise, clinician guidance, patient co-morbidity, and patient preferences.

The guidelines from the American Urological Association (AUA) and Société Internationale d'Urologie (SIU) recommend treatment with endoscopic techniques for first time bulbar strictures [1]. For recurrent strictures the guideline recommendations differ, with the AUA recommending urethroplasty and the SIU recommending endoscopic approaches *or* urethroplasty if symptomatic recurrence is after more than 3 months [1]. The reviews underpinning published guidelines, sponsored jointly by the SIU, the International Consultation on Urologic Disease [1, 5] and by the AUA [7], included non-randomised studies, predominantly of retrospective cohort design. Consequently the formulation of the guideline recommendation subsequent to these reviews was predominantly based on expert opinion and panel consensus rather than any robust evidence of the relative effectiveness or cost-effectiveness of these two treatment options.

To address this evidence gap, a randomised controlled trial (RCT) was conducted to compare the clinical and cost-effectiveness of urethroplasty versus endoscopic urethrotomy for the alleviation of urinary symptoms in men with recurrent bulbar stricture over 24 months (the OPEN (open urethroplasty versus endoscopic urethrotomy) Trial [6]). Clinical results of the OPEN trial are presented elsewhere [9]. This paper presents the cost-effectiveness analysis results.

## Methods

OPEN was a pragmatic patient-randomised two-arm superiority trial, which recruited across 38 National Health Service (NHS) secondary care providers in the UK. The algorithm allocated participants to each intervention in a 1:1 ratio with recruitment site and time since last procedure (< 12 months or ≥ 12 months) as minimisation covariates. Trial procedures and statistical analysis are described elsewhere [6, 9].

The OPEN trial’s economic analysis comprised a within trial cost-effectiveness analysis and Markov model with a 10-year time horizon. The within trial analysis estimated the costs from a societal perspective, quality adjusted life years (QALYs) gained and incremental cost per QALY of open urethroplasty compared to endoscopic urethrotomy over a 24-month period. As urethroplasty was a priori expected to be both more effective and more costly than endoscopic urethrotomy, with its benefits persisting beyond 24 months, Markov modelling [10] was conducted to examine the relative efficiency with a 10-year time horizon. Costs were reported as pound Sterling for the price year 2017. Where costs came from different years they were converted to 2017 values using the Consumer Price Index [11], as this was the last full year when unit costs were available before the analysis was conducted. All costs and QALYs were appropriately discounted using the recommended discount rate (3.5% per annum) [12]. Sensitivity analysis [13] was conducted to address uncertainty in study parameters and multiple imputation [14] was adopted to deal with missing data.

### Resource use and cost

Micro costing methods [15] were used to estimate the cost of the two interventions on a per patient basis. The cost of the interventions included the staff involved, the use of reusable and disposable equipment and use of the theatre suite. Data that varied by participant were collected on the trial’s Case Report Form, whilst other data came from communications with clinicians at the main trial site (Freeman hospital, Newcastle upon Tyne, UK). The unit costs of these resources came from standard sources [16–18].

When trial participants had a re-intervention during the trial’s follow-up period, the same process of micro costing used to cost the index procedure was invoked. Other use of primary and secondary NHS services, medications and participants’ out-of-pocket expenses relating to the condition came from the Case Report Form and a bespoke participant cost questionnaire completed at six-monthly intervals. Unit costs came from standard sources and participant cost questionnaire.

### Quality-adjusted life years

QALYs were based on responses to the EQ-5D-5L collected at baseline, immediately prior to surgery, 1 week after catheter removal, 3, 6, 9, 12, 24 months following surgery, 18 and 24 months after randomisation and at the end of study. The responses to the EQ-5D-5L questionnaire were scored using UK population tariffs [19] to produce a health state utility score for each participant in each of the treatment groups using the area under the curve (AUC) method [20].

Given the large number of time points for EQ-5D-5L data and to align with the primary effectiveness analysis [9], it was decided that to be included in the AUC analysis as a complete case, the participant must have at least three EQ-5D-5L observations with one at the start of the assessment period, one at the mid-range and one at the end. The specific requirements depended on whether the participants had an initial intervention and the type of analysis conducted (details in the note under Table 2).

For all calculations of QALYs, the first observation used was set at time point zero and the actual date of completion for each individual EQ-5D-5L questionnaire was used to calculate the number of days from the first observation. In the sensitivity analyses, QALYs were rescaled to the nominal data collection points, i.e. 730 days, to account for difference in waiting times between the two interventions (note that cost data did not require rescaling in the same way as the recall period, because it was pre-defined within the data collection tools). Additionally, multiple imputation for EQ-5D-5L at all missing time points was conducted to calculate QALYs for all participants.

### Within trial cost-utility analysis

The cost-utility analysis used an intention-to-treat principle. The incremental cost-effectiveness ratio (ICER) was calculated by dividing the difference in mean costs by the difference in mean QALYs for each group. Results were presented as point estimates of the mean incremental costs, QALYs and cost per QALY, estimated using seemingly unrelated regression [21], controlling for dichotomised time since last procedure (less than 12 months, 12 months or more), allocated treatment arm and baseline utility. Multiple imputation was performed to complete any missing data and used in the sensitivity analysis. Stochastic uncertainty in parameter estimates was addressed through the application of bootstrapping and the estimation of cost-effectiveness acceptability curves.

### Markov model

The Markov model consisted of three health states—symptom-free, symptomatic and deceased—in care pathways describing the process of care and disease incidence and progression. The base case analysis used parameters that were estimated based on information given by study participants who were allocated and received the allocated treatment. Both probabilistic and deterministic sensitivity analyses were conducted to address uncertainty. Deterministic sensitivity analyses included basing model parameters on those who received the same treatment procedure regardless of their allocated intervention group (due to cross-over in the trial) and varying the probabilities of follow-up intervention conditional on the previous intervention. The parameters used to populate the Markov model are reported in supplementary material (Additional file 1: Table S1). Model structure is shown in supplementary material (Additional file 1: Figure S3).

## Results

A total of 222 men were randomised, two of whom were excluded from analysis post-randomisation because further assessment prior to intervention found them to be ineligible. 108 were in the urethroplasty group and 112 in the urethrotomy group.

### Costs

Total costs combining NHS resource use costs (intervention, re-intervention and health service use during follow-up) and patients’ out-of-pocket costs are presented in Table 1. The cost of urethroplasty was statistically significantly higher over 24 months post randomisation than that of urethrotomy, with the cost difference ranging between £1333 and £2123 depending on whether follow-up care and patient costs were included for the base care and sensitivity analyses**.**

**Table 1:** Total cost (£) for each trial group

Total cost	Urethroplasty		Urethrotomy	
	Mean (SD) £	N	Mean (SD) £	N
*Base case*				
Total intervention and re-intervention cost	4332 (3151)	89	2209 (2368)	91
Total NHS cost (intervention and re-intervention with follow-up)	4455 (3191)	89	2657 (3476)	91
Total societal cost (NHS and patient costs)	4480 (3218)	89	2730 (3713)	91
*Sensitivity analysis with data imputation*				
Total intervention and re-intervention cost	4559 (3061)	108	2911 (2713)	112
Total NHS cost (intervention and re-intervention with follow-up)	4674 (3135)	108	3310 (3552)	112
Total societal cost (NHS and patient costs)	4704 (3155)	108	3371 (3755)	112

### Quality-adjusted life years

Estimates of QALYs for the base case analysis and all the sensitivity analyses are presented in Table 2. Urethrotomy in general appears to generate higher QALYs than urethroplasty. However, the difference was not statistically significant apart from evidence of a slight difference for the rescaled QALYs at 24 months after surgery (*p* < 0.1) and the rescaled QALYs with imputation (*p* < 0.1).

**Table 2:** Utility values at each time point and QALYs over the trial follow-up

EQ-5D 5L	Urethroplasty		Urethrotomy	
	Mean (SD)	N	Mean (SD)	N
QALYs at 24 months after randomisation	1.75 (0.40)	55	1.76 (0.35)	54
QALYs at 24 months after randomisation (rescaled to 730 days)	1.66 (0.34)	55	1.70 (0.34)	54
QALY at 24 months after surgery	1.73 (0.54)	44	1.77 (0.34)	56
QALY at 24 months after surgery (rescaled to 730 days)	1.42 (0.40)	44	1.58 (0.30)	56
QALY at 24 months after randomisation with imputation	1.73 (0.32)	108	1.76 (0.28)	112
QALY at 24 months after randomisation with imputation (rescaled to 730 days)	1.67 (0.29)	108	1.72 (0.27)	112
QALY at 24 months after surgery with imputation	1.75 (0.37)	108	1.76 (0.29)	112
QALY at 24 months after surgery with imputation (rescaled to 730 days)	1.67 (0.30)	108	1.72 (0.26)	112

*QALY calculations* For those participants who did not receive an initial intervention, to be included in the AUC analysis without imputation, they must have complete EQ-5D-5L data on all of the three time points: baseline, 18 months and 24 months after randomisation. For those participants who received an initial intervention, the base case analysis examined QALY over the period from baseline to 24 months after randomisation, therefore, the base case AUC analysis required complete EQ-5D-5L data at baseline and 24 months after randomisation, and at one of the data collection points of 3, 6, 9, 12 following surgery and 18 months following randomisation. Given the differences in the time lengths between randomisation and undergoing an intervention between urethroplasty and urethrotomy, sensitivity analyses also examined QALY over the period from the time prior to surgery to 24 month post-surgery, in which case the AUC analysis required complete EQ-5D-5L data at prior to surgery and 24 months after surgery, and at one of the data collection points of 3, 6, 9, 12 following surgery and 18, 24 months following randomisation

### Within trial cost-utility analysis

**Fig. 1:**
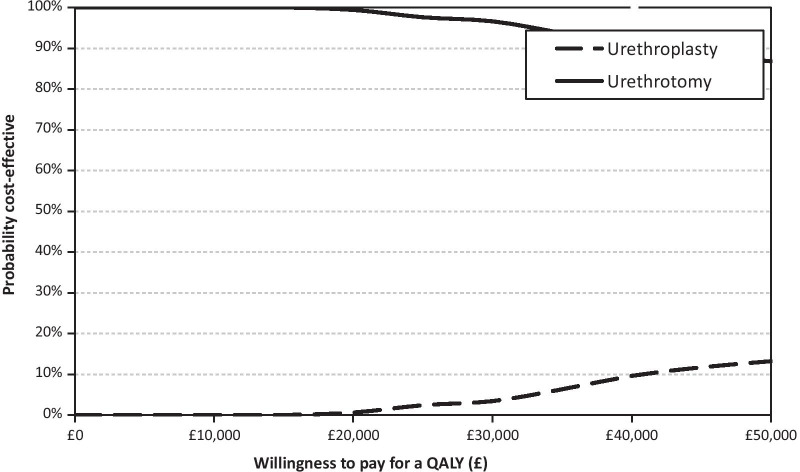
Cost-effectiveness acceptability curve (Base case)

In the base case, urethroplasty costed more than urethrotomy while generating a lower QALY, therefore, was dominated by urethrotomy (Table 3). The base case results appeared robust as they were similar in the sensitivity analyses (Table 3). The cost-effectiveness acceptability curve (Fig. 1) and incremental cost and QALY plots (Additional file 1: Figure S1) are presented for the base case.

**Table 3:** Cost-effectiveness analysis (within trial)

Investigation strategy	Cost (£) mean (95% CI)	Incremental Cost (£) mean (95% CI)	QALY mean (95% CI)	Incremental effect mean (95% CI)	ICER (£)	Probability of each treatment strategy is cost-effective for different threshold values for society’s willingness to pay				
						£0 k (%)	£10 k (%)	£20 k (%)	£30 k (%)	£50 k (%)
*Base case, 24 months post randomisation*										
Urethroplasty (n = 46)	4869 (4123, 5614)	2148 (689, 3606)	1.74 (1.61, 1.86)	− 0.01 (− 0.17, 0.14)		0	0	1	3	13
Urethrotomy (n = 46)	2721 (1444, 3999)		1.75 (1.65, 1.85)		Dominant	100	100	99	97	87
*24 months post randomisation (rescaled QALY)*										
Urethroplasty (n = 46)	4869 (4123, 5614)	2148 (689, 3606)	1.65 (1.55, 1.76)	− 0.04 (− 0.18, 0.11)		0	0	0	1	4
Urethrotomy (n = 46)	2721 (1444, 3999)		1.69 (1.59, 1.79)		Dominant	100	100	100	99	96
*24 months post surgery*										
Urethroplasty (n = 37)	4963 (3977, 5949)	1672 (− 65, 3409)	1.73 (1.54, 1.92)	− 0.04 (− 0.24, 0.16)		0	0	4	9	15
Urethrotomy (n = 48)	3291 (1947, 4636)		1.77 (1.67, 1.87)		Dominant	100	100	96	91	85
*24 months post surgery (rescaled QALY)*										
Urethroplasty (n = 37)	4963 (3977, 5949)	1672 (− 65, 3409)	1.42 (1.28, 1.56)	− 0.16 (− 0.31, − 0.01)		0	0	0	0	0
Urethrotomy (n = 48)	3291 (1947, 4636)		1.58 (1.49, 1.67)		Dominant	100	100	100	100	100
*24 months post randomisation with imputation*										
Urethroplasty (n = 108)	4704 (4102, 5305)	1333 (410, 2256)	1.73 (1.67, 1.79)	− 0⋅03 (− 0.11, 0.05)		0	0	0	0	1
Urethrotomy (n = 112)	3371 (2667, 4074)		1.76 (1.71, 1.81)		Dominant	100	100	100	100	99
*24 months post randomisation with imputation (rescaled QALY)*										
Urethroplasty (n = 108)	4704 (4102, 5305)	1333 (410, 2256)	1.67 (1.62, 1.73)	− 0.05 (− 0.13, 0.02)		0	0	0	0	0
Urethrotomy (n = 112)	3371 (2667, 4074)		1.72 (1.67, 1.77)		Dominant	100	100	100	100	100
*24 months post surgery with imputation*										
Urethroplasty (n = 108)	4704 (4102, 5305)	1333 (410, 2256)	1.75 (1.68, 1.82)	− 0.02 (− 0.10, 0.07)		0	0	0	0	3
Urethrotomy (n = 112)	3371 (2667, 4074)		1.76 (1.71, 1.82)		Dominant	100	100	100	100	97
*24 months post surgery with imputation (rescaled QALY)*										
Urethroplasty (n = 108)	4704 (4102, 5305)	1333 (410, 2256)	1.67 (1.61, 1.73)	− 0.05 (− 0.12, 0.02)		0	0	0	0	0
Urethrotomy (n = 112)	3371 (2667, 4074)		1.72 (1.67, 1.77)		Dominant	100	100	100	100	100

### Markov model

**Fig. 2:**
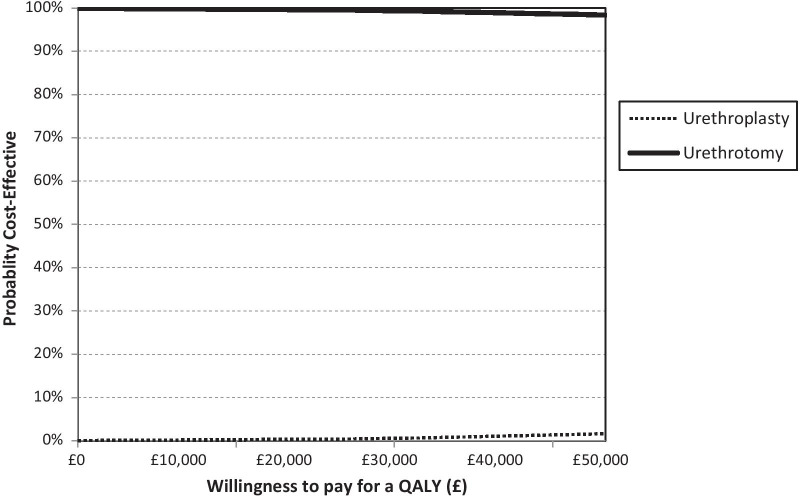
Cost-effectiveness acceptability curve (Markov model base case)

In the base case analysis, urethroplasty is unlikely to be considered cost-effective under current society’s willingness to pay threshold for a QALY and this is supported by the sensitivity analyses results (Table 4 and Figs. 2 and Additional file 1: Figure S2). This is mainly due to the higher cost of urethroplasty compared to urethrotomy, whilst both of the treatment options produce similar QALY gains. This is despite those receiving urethroplasty having a lower chance of recurrence compared to those receiving urethrotomy.

**Table 4:** Markov model result over 10 years

Analyses	Treatment strategy	Cost (£)	QALY	ICER (£)	Probability of each treatment strategy is cost-effective for different threshold values for society’s willingness to pay for a QALY				
					£0 k (%)	£10 k (%)	£20 k (%)	£30 k (%)	£50 k (%)
Base case	Urethroplasty	8026	7.61	301,073	0	0	0	0	2
	Urethrotomy	6553	7.60		100	100	100	100	98
Parameters based on treatment received	Urethroplasty	7987	7.61	307,328	0	0	0	0	1
	Urethrotomy	6490	7.60		100	100	100	100	99
Always receive the same treatment at recurrence	Urethroplasty	9026	7.61	476,162	0	0	0	0	0
	Urethrotomy	4059	7.60		100	100	100	100	100
Always receive the other treatment at recurrence	Urethroplasty	8076	7.61	263,383	0	0	1	2	4
	Urethrotomy	7054	7.60		100	100	99	98	96

## Discussion

Relative efficiency within the trial follow-up and over a 10-year time horizon was found to favour urethrotomy compared with urethroplasty. Effectiveness as measured by QALYs appeared to be broadly equivalent between the two trial arms of the OPEN trial, and this was in line with the primary clinical outcome finding showing no statistically significant difference in the primary patient reported outcome between trial arms [9]. Costs were higher in the urethroplasty arm than the urethrotomy arm. Therefore, urethroplasty had a higher cost whilst producing similar QALY gain, making it unlikely to be cost-effective, despite having a lower chance of recurrence compared to urethrotomy.

The relatively high cost of urethroplasty is the main reason that it was not as cost-effective. It may be possible to reduce the cost of urethroplasty by using day-care surgery [22], a shorter period of catheterisation [23] or by rationalising follow-up to those most likely to need it and discharging symptom stable patients at an early stage [24]. Length of stay following urethroplasty within the trial was 1.34 (SD 0.95) days vs 0.52 (SD 1) for urethrotomy [25]. Therefore, scope to substantially reduce length of stay is limited and even if length of stay was reduced to zero this would be insufficient to reduce the cost of urethroplasty to close to the cost of urethrotomy.

However, the higher cost of urethroplasty is driven by the higher theatre procedure and it is unlikely that theatre time could be reduced significantly to alleviate the increased costs of urethroplasty. Hospitalisation accounts for around 15% of the total intervention cost. Strategies to reduce length of stay could be explored as a way of lowering the cost of urethroplasty. In Europe, practice varies widely with length of stay ranging from 2 to 7 days in high volume centres exaggerating the difference between the two procedures costs. The mean length of stay in the UK trial was 2 days. It is unlikely that further substantial reductions in length of stay would be possible, although pre-operative counselling, improved perioperative analgesia and discharge education could help reduce this to 1.5 days.

Although the study showed no difference in effectiveness as measured by QALYs, it is worth noting that the loss of quality of life during recovery from subsequent procedures was not taken into consideration. Given the likelihood of recurrence requiring repeated treatments, this decrement in quality of life could make a difference in total QALYs between the two treatments. Patients undergoing urethroplasty are also less likely to have recurrence in the long-term based on the observation during the trial’s follow-up period and consequently suffer less loss in quality of life and hence may accrue more QALYs over time. However, there was no obvious signal of reductions in quality of life caused by differences in recurrent rates [25]. However, this requires further study. Additionally, EQ-5D as a generic quality of life measure may not be sensitive enough to capture changes in health-related quality of life among patients with bulbar urethral stricture, as the impact on quality of life from the condition may fall mostly around the time of recurrence or only present in one or two dimensions (e.g. pain, anxiety) of EQ-5D. Future research should focus on how best to capture quality of life loss during recurrence and exploring alternative measures for this type of patients using preference elicitation methods [26, 27].

When examining the cost-effectiveness over the long-term, a key uncertainty in the modelling was the choice of re-intervention. The study data showed a large proportion of patients switched to a treatment different from their previous treatment when they had a re-intervention. There is no consensus on treatment choices for re-interventions, and such choices are often influenced by many non-clinical factors such as patient choice, waiting time, and travel time [8]. However, varying the probabilities of treatment options conditional on the previous intervention in the sensitivity analyses conducted for the modelling showed that urethroplasty remained less likely to be cost-effective.

Due to the trial’s follow-up period of two years, we could potentially be underestimating the costs of the urethrotomy arm, however, questions arise on how many recurrences there need to be to offset the more expensive urethroplasty and over what length of time. On the other hand, there was little evidence suggesting patients’ quality of life differed significantly between the two arms, at least within the trial’s follow-up period. We do not claim that cost is the only concern in the choice of treatment, but in the absence of clear evidence on the additional benefits one treatment can bring, costs may be an important factor to consider in a publicly funded health system. These issues were also extrapolated over 10 years using a Markov model, which met or exceeded internationally accepted guidelines for best practice for the conduct of such work. This analysis showed that the initial higher costs of urethroplasty were not likely to be offset by increased reintervention rates but that urethroplasty was on average more effective.

The key strength of the present study is the use of an RCT designed to detect the clinically meaningful difference in voiding symptom score. The RCT design was based on current best practice for pragmatic surgical trials and it sought to provide direct and most up to date information on costs and utilities. However, obtaining data directly from a large clinical study also has its downside. The principle limitation of the RCT was the missing data caused by trial participants being lost to follow-up; a common situation in a complex study involving several years of follow-up. We remedied this by using multiple imputation for the missing data and comparing complete case analysis with analyses using imputation. These sensitivity analyses demonstrated that the study results were robust.

Although the economic evaluation was conducted in the UK NHS, the results are generalisable to other countries with similarly public funded healthcare systems. As we have seen that there is little difference in QALYs between the two treatment options, regardless of unit costs of resources used, urethroplasty is generally more costly than urethrotomy due to the longer operating and recovery time. Therefore, the conclusions drawn for QALYs and costs would stay unchanged in other settings.

## Conclusions

This paper has presented a comprehensive economic analysis of the relative efficiency of urethroplasty and urethrotomy within an RCT over 24 months and over a 10-year time horizon. Based on current practice and evidence, urethroplasty is unlikely to be cost-effective due to its higher cost. Future research should examine how the benefits of reduced recurrence by urethroplasty can be captured while finding ways to reduce its costs.

## Supplementary Information

**Additional file 1.** Additional material on the methods and results of the economic evaluation.

## Data Availability

The data used in the analysis are not publically available due to data protection, but anonymised data can be made available from corresponding author upon reasonable request.

## Abbreviations

OPEN: Open urethroplasty versus endoscopic urethrotomy
NHS: National Health Service
QALY: Quality adjusted life years
AUC: Area under the curve
ICER: Incremental cost-effectiveness ratio
RCT: Randomised controlled trial
