# Supplementary material for: Endoscopic urethrotomy versus open urethroplasty for men with bulbar urethral stricture: the OPEN randomised trial cost-effectiveness analysis
Source: BMC Urol. 2021 May 3;21:76. doi: 10.1186/s12894-021-00836-1 (PMC8094457; doi:10.1186/s12894-021-00836-1)

**Endoscopic urethrotomy vs open urethroplasty for men with bulbar urethral stricture: The OPEN randomised trial cost-effectiveness analysis**

**Authors:** Jing Shen^1^, Luke Vale^1*^, Beatriz Goulao^2^, Paul Whybrow^3^, Stephen Payne^4^, and Nick Watkin^5^ for the OPEN trial investigators

1. Population Health Sciences Institute, Newcastle University, Newcastle upon Tyne, UK

2. Health Services Research Unit, University of Aberdeen, Aberdeen, UK

3. Hull York Medical School, University of Hull, Hull, UK

4. Central Manchester Hospitals NHS Foundation Trust, Manchester, UK

5. Department of Urology, St George’s University of London, London, UK

*corresponding author (luke.vale@newcastle.ac.uk)

Supplementary materials

Table S1: Model parameters

| **Parameter name** | **Value** | **Source and distribution** |
| --- | --- | --- |
| Cost of urethroplasty (including initial surgery with catheter removal and hospital stay) | Base case - £5871 (£229)  By treatment received - £5808 (£219) | Based on value from the trial; Gamma distribution; |
| Cost of urethrotomy (including initial surgery with catheter removal and hospital stay) | Base case - £1316 (£96)  By treatment received - £1367 (£90) | Based on value from the trial; Gamma distribution; |
| Utility associated with cured (symptom free) health state | Base case – 0.899 (0.013)  By treatment received – same as base case | Based on value from the trial; Beta distribution; utility values at 3 months after surgery of those who showed a significant improvement in voiding scores. |
| Utility associated with symptomatic health state | Base case – 0.852 (0.014)  By treatment received – same as base case | Based on value from the trial; Beta distribution; Utility values at baseline |
| Cost of health service use and patient’s out of pocket expenses following urethroplasty | Base case - £130 (£46)  By treatment received - £141 (£45) | Based on value from the trial; Gamma distribution; |
| Cost of health service use and patient’s out of pocket expenses following urethrotomy | Base case - £227 (£42)  By treatment received - £210 (£38) | Based on value from the trial; Gamma distribution; |
| Surgery success rate for urethroplasty | Base case - 0.95 (0.03)  By treatment received – 0.94 (0.03) | Based on value from the trial; Beta distribution; |
| Surgery success rate for urethrotomy | Base case - 0.91 (0.03)  By treatment received – 0.92 (0.03) | Based on value from the trial; Beta distribution; |
| Probability of being treated when symptomatic | Base case - 0.90 (0.02)  By treatment received – same as base case | Based on value from the trial; Beta distribution; |
| Probability of receiving urethroplasty if the last treatment is urethroplasty | Base case - 0.12 (0.12)  By treatment received – 0.11 (0.11) | Based on value from the trial; Beta distribution; |
| Probability of receiving urethroplasty if the last treatment is urethrotomy | Base case - 0.70 (0.08)  By treatment received – 0.63 (0.07) | Based on value from the trial; Beta distribution; |
| Probability of recurrence following urethroplasty | Base case – 0.042  By treatment received – 0.041 | Based on survival analysis from the trial; |
| Probability of recurrence following urethrotomy | Base case – 0.1497  By treatment received – 0.150 | Based on survival analysis from the trial; |

Figure S1: Incremental costs and QALY plots (Base case)


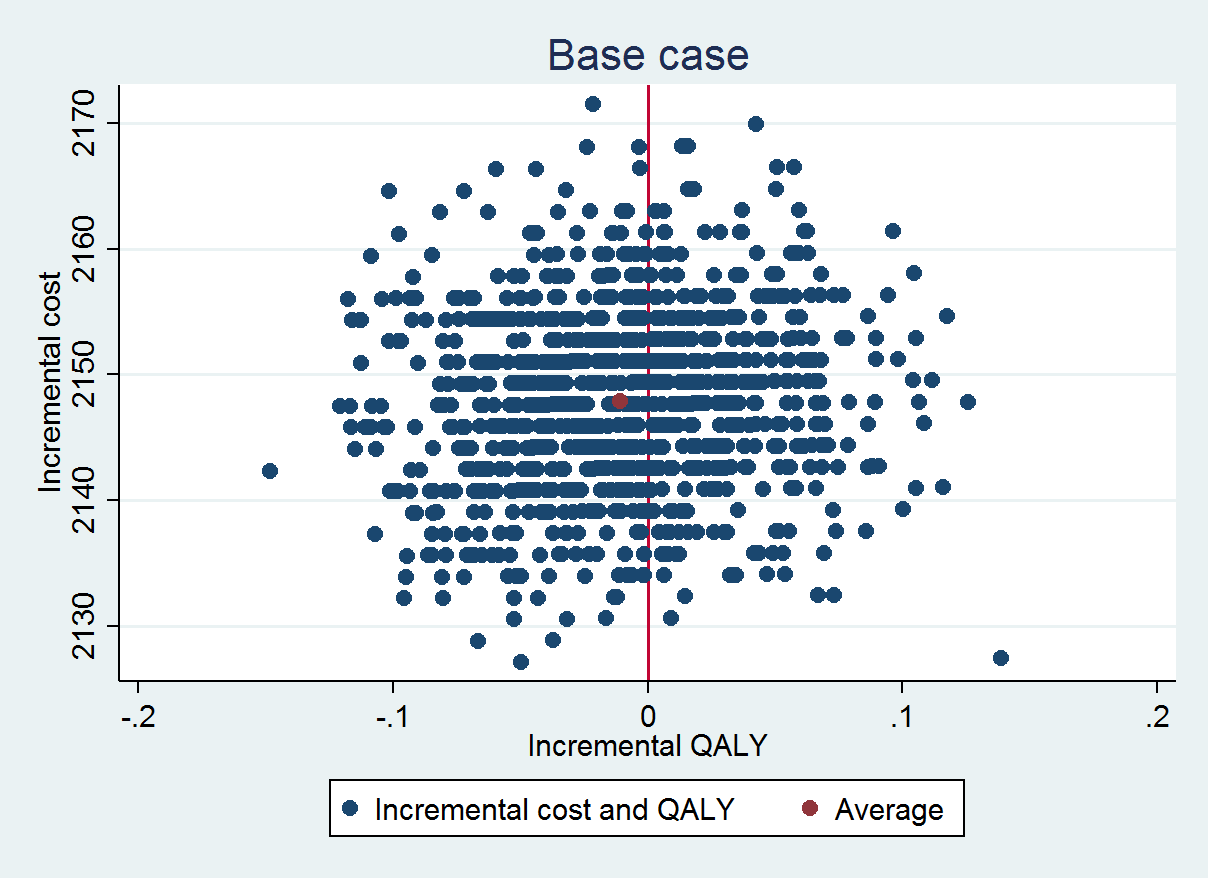


Figure S2: Incremental costs and QALY plots (Markov Model base case)

Figure S3, Markov model structure


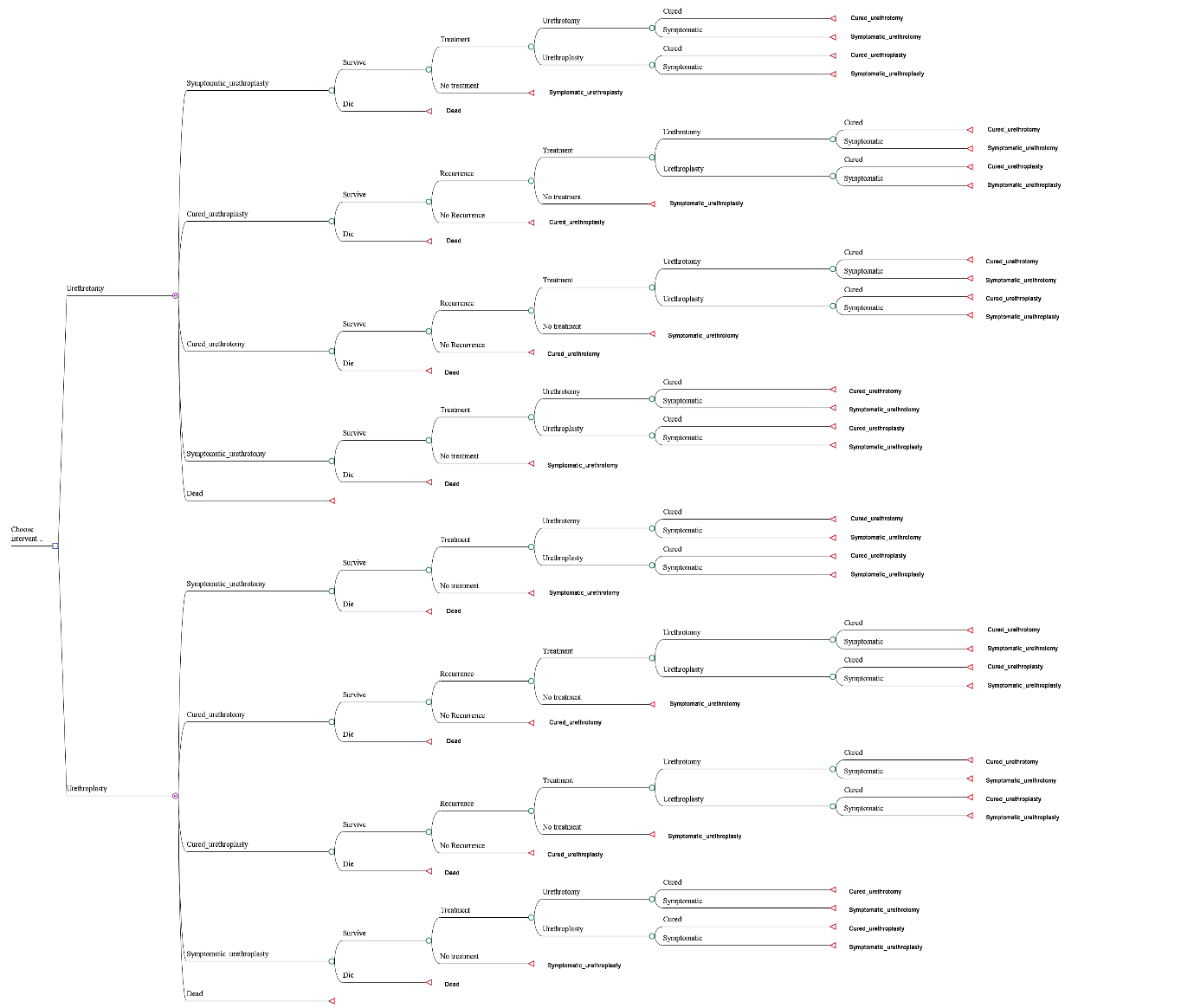

Supplement: Supplementary file 1 — Additional file 1. Additional material on the methods and results of the economic evaluation. [file 12894_2021_836_MOESM1_ESM.docx]
